# Early, Delayed and Late Cardiac Implantable Electronic Device Infections: Do the Timing of Onset and Pathogens Matter?

**DOI:** 10.3390/jcm11143929

**Published:** 2022-07-06

**Authors:** Anna Polewczyk, Wojciech Jacheć, Maciej Polewczyk, Dorota Szczęśniak-Stańczyk, Andrzej Kutarski

**Affiliations:** 1 Department of Physiology, Pathophysiology and Clinical Immunology, Collegium Medicum of Jan Kochanowski University, 25-369 Kielce, Poland; 2 Department of Cardiac Surgery, Świętokrzyskie Cardiology Center, 25-736 Kielce, Poland; 3 2nd Department of Cardiology, Zabrze, Faculty of Medical Science in Zabrze, Medical University of Silesia in Katowice, 40-055 Katowice, Poland; wjachec@interia.pl; 4 Department of Microbiology, Collegium Medicum of Jan Kochanowski University, 25-317 Kielce, Poland; maciek.polewczyk@gmail.com; 5 Acute Cardiac Care Unit, Świętokrzyskie Cardiology Center, 25-736 Kielce, Poland; 6 Department of Cardiology, Medical University, 20-059 Lublin, Poland; dorotastanczyk@gmail.com (D.S.-S.); a_kutarski@yahoo.com (A.K.)

**Keywords:** cardiac implantable electronic device related infection, pocket infection, lead-related infective endocarditis, time to infection onset, pathogens

## Abstract

Infections involving cardiac implantable electronic devices (CIEDs) occur at different times after device-related procedures. The aim of this study was to investigate the timing of onset and factors influencing the occurrence of all types of CIED infections to identify the type of pathogen and to examine the long-term survival of patients with all types of CIED infections. We performed a post hoc analysis of the clinical data from 3344 patients who underwent transvenous lead extraction (TLE) at a single high-volume center between 2006 and 2020, including a group of 890 patients with CIED infections. The occurrence of pocket infection (PI), lead-related infective endocarditis (LRIE) and PI coexisting with LRIE (PI + LRIE) was assessed at the following time intervals: 0–12 months, 13–36 months and > 36 months since last CIED-related procedure. In the study group, there were 274 (30.79%) early infections, 266 (29.89%) delayed infections and 350 (39.32%) late infections. Pocket infection was the most common early complication (97; 39.43%), while LRIE was predominant over 36 months from the last CIED procedure (172; 54.09%). The most common early infections were PIs that were associated with the preceding CIED-related procedure. Late LRIE was most likely to occur in patients with intracardiac lead abrasion. The probability of early versus late LRIE was higher in patients with CoNS cultures. The timing of infection onset irrespective of its type does not affect long-term survival after transvenous lead extraction. The majority of infectious complications (69%) occur more than 12 months after the last CIED-related procedure. Early infections are probably associated with pocket contamination during CIED-related procedure, while delayed and late systemic infections are related to other lead-dependent factors (especially to intracardiac lead abrasion). Time to LRIE onset is associated with pathogen type. The timing of symptom onset does not affect long-term survival after TLE.

## 1. Introduction

Infectious complications related to cardiac implantable electronic devices (CIEDs) occur in 2.3–3.4% of CIED recipients [1,2,3]. Depending on the severity of the inflammatory process, there are three types of CIED infections: pocket infection (PI), lead-related infective endocarditis (LRIE) and PI coexisting with LRIE. Prior studies [4,5] have shown that the most common cause of LRIE is the spread of infection from the generator along the leads to the endocardium. The analysis of the time relationship of infectious complications with the last CIED-related procedure may suggest the existence of a different mechanism involved in the pathogenesis of the different types of infections. Previous reports also suggested that early and late infections may be caused by different microorganisms, but research has been inconclusive. According to several studies, the most common pathogens causing both early and late infections are Staphylococcal species [6]. Another report found that there were more non-Staphylococcal infections in the late group compared to the early group [7], whereas, according to yet another study, Staphylococcus aureus was more likely to cause early than late infections [8]. In the present study, we assessed the occurrence of PI, PI + LRIE and LRIE at specified time intervals after the last revision procedure, and we also analyzed the types of pathogens and the long-term survival rate of patients with infectious complications.

## 2. Material and Methods

### 2.1. Study Population

We performed a post hoc analysis of data from 3344 patients who underwent transvenous lead extraction (TLE) at a high-volume center (consisting of 3 branches in Lublin, Zamość, Radom in Poland) between 2006 and 2020. The clinical records from 890 (28.58%) patients with CIED infections were analyzed including 246 (27.64%) PI, 318 (35.73%) LRIE and 326 (36.63%) PI + LRIE cases. A total of 178 (24.78%) cases of possible endocarditis were included in the LRIE and PI + LRIE groups.

### 2.2. Analysis of Infectious Complications in Specific Time Intervals

Infectious complications were divided into: early—occurring within 0–12 months after preceding CIED-related procedure; delayed—appearing in the period from 13 months to 36 months; and late—found over 36 months after revision procedure. Factors potentially associated with the occurrence of PI, LRIE and PI + LRIE were analyzed at each time interval taking into account the clinical data (age, gender, concomitant diseases) and the device-related factors (type of system, number of leads, lead dwell time, lead-related abnormalities, procedures before TLE), as well as long-term survival after TLE (mean follow-up 1826 ± 1381 days). A microbiological analysis of cultures from device pockets, extracted leads and blood was also performed, assessing the type of pathogens at each time interval. Cultures from the removed leads were obtained only from patients without local pocket infection.

### 2.3. Definitions

A pocket infection was defined as the presence of local warmth, erythema, edema and pain or discharge from the device pocket or an erosion or impending erosion of the device according to the European Society of Cardiology (ESC) guidelines [9].

Lead-related infective endocarditis was diagnosed using modified Duke criteria according to the 2015 ESC guidelines for the management of infective endocarditis [9] and the European Heart Rhythm Association international consensus document [10]. The diagnosis of LRIE was definite in the presence of two major criteria or one major criterion and three minor criteria. Possible LRIE was diagnosed when the presence of one major criterion and one minor criterion or three minor criteria was confirmed.

Transvenous lead extraction procedure was defined according to the Heart Rhythm Society (HRS) and the European Heart Rhythm Association (EHRA) statements [11,12,13].

The term CIED-related procedure (revision procedure) was used to define the following types of procedures performed before infection: generator replacement, system upgrading or downgrading, placement of an additional lead and prior transvenous lead extraction.

Intracardiac lead abrasion was defined as outer insulation macroscopic damage, located in the intracardiac portion of the lead, usually in the first 15–20 cm from the tip, with visible discoloration, frequently with conductor externalization and often with purulent discharge [14].

### 2.4. Statistical Analysis

Distribution of all continuous variables was evaluated by the Shapiro–Wilk test. The distribution was non-parametric. More of them were expressed as means with standard deviation, wherein values of NYHA FC class and number of previous CIED-related procedures were expressed as median with IQR. All were compared with the ANOVA Kruskal–Wallis test. If there was a statistically significant difference in ANOVA Kruskal–Wallis test, the Mann–Whitney U test was used to compare all individual variables with each other. Categorical data are presented as absolute numbers and percentages and were compared using the Kruskal–Wallis ANOVA test. If there was a statistically significant difference in the Kruskal–Wallis ANOVA test, the χ^2^ test with Yates correction was used to compare all individual variables with each other.

The patients were subdivided into groups based on temporal onset and type of infection related to last CIED procedure for comparison within the disease entity and between groups at a given time interval. Characteristics are marked with capital letters E (early), D (delayed) or L (late) in the case of statistically significant differences within the disease entity or Arabic numerals in the case of statistically significant differences between disease entities. Cox regression analysis was performed to identify the variables associated with infection. The survival time was defined as the period elapsing between the last CIED-related procedure (including initial CIED implantation) for noninfectious indications and TLE. The analysis was performed for each infectious complication (PI, PI + LRIE and LRIE) separately at each time interval (0–12. 13–36. >36 months): 0–12 months—data from all the 3114 patients were included; 13–36 months—patients who reached the study endpoints (infection) 12 months since last CIED-related procedure, but patients with noninfectious indications for TLE with follow-up <12 months were excluded from analysis; >36 months—patients who reached the study endpoints (infection) 36 months since last CIED-related procedure, but patients with noninfectious indications for TLE with follow-up <36 months were excluded from analysis.

To determine which factors have an impact on time to infection onset, univariable linear logistic regression was used to evaluate all the data from Table 1 and Table 2. The variables having *p* values < 0.05 in univariable regression analysis were included in multivariable analysis. Logistic regression analysis included the data from patients with infection occurring 12 months and >36 months since last CIED-related procedure for noninfectious indications. 

For each type of infectious complication, the Kaplan–Meyer curves were used to analyze the event-free survival between groups depending on temporal onset of infection, and the differences were tested for significance by the log-rank test.

A *p* value < 0.05 was considered statistically significant. Lack of statistical significance was described as NS (nonsignificant).

Statistical analysis was performed using STATISTICA 13.1 PL (TIBCO Software Corp., Cracow, Poland).

## 3. Results

**Figure 1:**
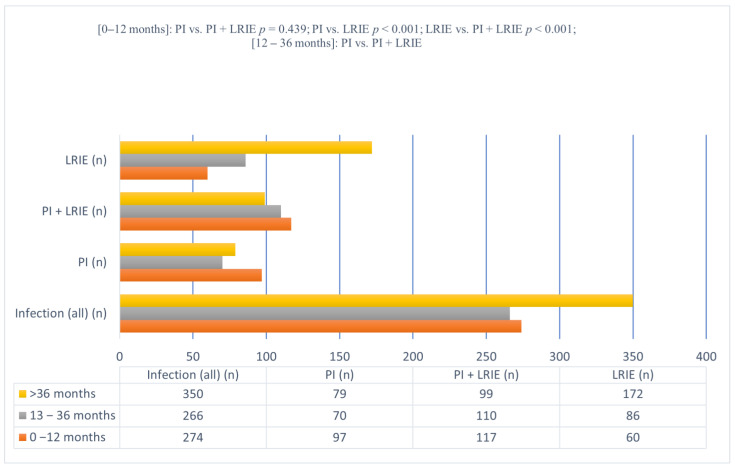
Prevalence of CIED infections at each time interval after a CIED-related procedure for noninfectious indications.

In the population of 890 patients who underwent transvenous lead extraction due to CIED infection, there were 274 (30.79%) early infections (0–12 months since last CIED-related procedure), 266 (29.89%) delayed infections (13–36 months) and 350 (39.32%) late infections (>36 months). At 12 months after a CIED procedure, the distribution of the infectious complications was as follows: 97 (39.43%) PI, 117 (35.89%) PI + LRIE and 60 (18.87%) LRIE (*p* < 0.001 vs. PI and vs. PI + LRIE). More than 50% of PI, more than 60% of PI + LRIE and more than 80% of LRIE occurred at a later time after a CIED-related procedure (more than 12 months). Late isolated lead-related infective endocarditis was the dominant subgroup of all CIED infections (172 cases). The fewest LRIE cases were found in the early infection group, while the highest number was in the late infection group (54.09%). The opposite tendency was seen in patients with isolated pocket infection and with PI + LRIE; in these groups, device infection most commonly occurred 12 months since last CIED procedure (Table 1, Figure 1).

**Table 1:** Prevalence of CIED infections at each time interval after a CIED-related procedure for noninfectious indications.

	Number of Cases	0–12 Months	13–36 Months	>36 Months
Infection (all); n (%)	890 (100.0%)	274 (30.79%)	266 (29.89%)	350 (39.32%)
Isolated pocket infection (PI); n (%)	246 (100.0%)	97 (39.43%)	70 (28.46%)	79 (32.11%)
Pocket infection with concomitant lead-related infective endocarditis (PI + LRIE); n (%)	326 (100.0%)	117 (35.89%) (*p* = 0.439 vs. PI)	110 (33.74%) (*p* = 0.209 vs. PI)	99 (30.37%) (*p* = 0.722 vs. PI)
Isolated lead-related infective endocarditis (LRIE); n (%)	318 (100.0%)	60 (18.87%) (*p* < 0.001 vs. PI; *p* < 0.001 vs. PI + LRIE)	86 (27.04%) (*p* = 0.782 vs. PI; *p* < 0.034 vs. PI + LRIE)	172 54.09% (*p* < 0.001 vs. PI; *p* < 0.001 vs. PI + LRIE)

CIED—cardiac implantable electronic device, LRIE—lead related infective endocarditis, PI—pocket infection.

Detailed analysis of clinical factors in individual types of infection in the studied time intervals showed that late LRIEs were more common in younger patients and in patients with heart failure with a higher NYHA class compared to late PI and late PI + LRIE (2 (IQR 1–3) vs. 2 (IQR 1–2) vs. 2 (IQR 1–2); *p* = 0.006).

There were significantly more women in the delayed LRIE group compared to the delayed PI and PI + LRIE groups (*p* = 0.044). Patients with LRIE more often had diabetes (27.36% vs. 22.92 vs. 22.70 in the PI and PI + LRIE groups; *p* = 0.013) and higher creatinine levels compared to the PI and PI + LRIE groups (1.59 ± 1.29 vs. 1.38 ± 1.09 vs. 1.29 ± 1.00; *p* < 0.001). These differences were significant in all analyzed time intervals. 

Detailed analysis of the remaining clinical factors revealed non-specific differences between the different types of infection in the time periods studied.

With regard to device-related factors and type and timing of infection, early infections, compared to late ones, were more common in patients with high voltage (HV) lead—significant differences were found in both the PI and LRIE groups (32.99% vs. 17.72%, *p* = 0.009, and 41.67% vs. 26.74%, *p* = 0.026, respectively). Early, delayed and late LRIEs were more common in patients with the presence of lead in the coronary sinus.

Time-dependent analysis showed a greater number of leads in patients with early infections, especially in early PI and early PI + LRIE compared to late infections of this type (*p*= 0.026 and *p* < 0.001, respectively). Similarly, there was a tendency for early infection in patients with abandoned leads, especially in the early PI + LRIE group compared to delayed and late infections of this type (27.35% vs. 14.55% vs. 8.08%; *p* < 0.001). 

The number of previous device-related procedures was lowest in patients with LRIE (1.79 ± 1.11 vs. 1.98 ± 1.13 in the PI + LRIE group vs. 2.09 ± 1.14 in the PI group; *p* = 0.004). Previous CIED-related procedures were on average significantly more common in the early PI rather than early LRIE group (2 (IQR 2–3) vs. 2 (IQR 1–2); *p* = 0.002). Moreover, in all types of infections, the number of revision procedures was lower in patients with late rather than early infections.

Dwell time of the oldest lead per patient during the last procedure before TLE was shorter in patients with late infections compared to early infections of all types. Late PI and late LRIE more often occurred in patients with longer lead dwell time during TLE compared to early infections of this type (113.7 ± 73.77 vs. 89.59 ± 58.71 months; *p* < 0.001 and 104.3 ± 61.07 vs. 67.33 ± 64.63 months; *p* < 0.001). 

Patients with late LRIE were more likely to have excessively elongated loops of the leads (9.30% vs. 5.06% in patients with late PI vs. 3.03% in patients with late PI + LRIE; *p* = 0.006). Similarly, the phenomenon of abrasion of leads was more often found in delayed and late LRIE compared to late PI and late PI + LRIE (33.72% vs. 27.14% vs. 12.73%; *p*= 0.002). 

Late infections of all types, especially late LRIE, were less likely to be associated with the preceding revision procedure (65.12% late LRIE occurred after primary implantation of CIED, without any preceding procedure), while the most early infections, especially early PIs (59.79%) were found after unit replacement (Table 2). 

**Table 2:** Clinical and procedural findings depending on type of infection and temporal onset related to last CIED procedure for noninfectious indications.

			Early (E)	Delayed (D)	Late (L)	ANOVA Kruskal-Wallis 3–5		Early (E)	Delayed (D)	Late (L)	ANOVA Kruskal-Wallis 7–9		Early (E)	Delayed (D)	Late (L)	ANOVA Kruskall-Wallis				
	1	2	3	4	5		6	7	8	9		10	11	12	13	11 12 13	2 6 10	3 7 11	4 8 12	5 9 13
	All	PI (All)	0–12 month	13–36 month	>36 month	*p*	PI +LRIE (All)	0–12 month	13–36 month	>36 month	*p*	LRIE (All)	0–12 month	13–36 month	>36 month	*p*	*p*	*p*	*p*	*p*
Time to TLE since last CIED procedure [months], (mean ± SD)	35.73 ±33.03	31.46 ±31.34	5.84 ±3.55	23.94 ±7.94	69.59 ±26.05		28.81 ±27.41	5.54 ±3.62	22.64 ±7.44	63.17 ±23.09	<0.001	46.13 ±36.85	6.53 ±3.38	23.24 ±7.07	71.38 ±32.14	<0.001	<0.001	0.200	0.447	0.062
	Patient characteristics																			
Number of patients; n (%)	890 (100)	246 (100.0)	97 (39.43)	70 ^E^ (28.46)	79 (32.11)		326 (100.0)	117 (35.89)	110 (33.74)	99 (30.37)		318 (100.0)	60 (18.87)	86 ^E^ (27.04)	172 ^DDD EEE^ (54.09)					
Patient age during last procedure before TLE [years]	65.20 ±14.31	67.19 ±13.39	68.88 ±13.83	66.64 ±13.79	65.61 ±12.36	0.079	66.66 ±13.79	66.81 ±13.77	66.59 ±15.40	66.57 ±11.94	0.665	62.18 ^222 666^ ±14.98	63.76 ^3^ ±15.41	64.53 ±13.56	60.46 ^D 5 99^ ±15.38	0.052	<0.001	0.061	0.278	0.004
Female patient; n (%)	275 (30.90)	72 (29.27)	37 (38.14)	11 ^E^ (15.71)	24 (30.38)	0.007	99 (30.37)	41 (35.04)	32 (29.09)	26 (26.26)	0.354	104 (32.70)	19 (31.67)	29 ^4^ (33.72)	56 (32.56)	0.965	0.426	0.786	0.044	0.518
NYHA [class]; (median, IQR)	2 [1–2]	2 [1–2]	2 [1–2]	2 [1–2]	2 [1–2]	0.992	2 [1–2]	2 [1–2]	2 [1–2]	2 [1–2]	0.991	2 ^2 66^ [1–3]	2 [1–2]	2 [1–2]	2 ^5 99^ [2–3]	0.288	0.002	0.689	0.491	0.006
LVEF [%]; (mean±SD)	47.92 ±14.74	48.57 ±14.23	48.49 ±14.10	46.19 ±14.23	51.0 ^D^ ±14.19	0.079	47.72 ±14.50	48.34 ±14.99	46.09 ±14.55	48.79 ±13.95	0.355	47.64 ±15.36	47.72 ±14.77	45.18 ±15.96	48.68 ±15.21	0.270	0.626	0.939	0.958	0.543
AF permanent; n (%)	234 (26.29)	77 (31.30)	28 (28.87)	23 (32.86)	26 (32.91)	0.803	77 (23.62)	29 (24.79)	22 (20.00)	26 (26.26)	0.531	80 (25.16)	12 (20.00)	20 (23.26)	48 (27.91)	0.428	0.107	0.461	0.114	0.502
Diabetes (any) n (%)	204 (22.92)	43 (17.48)	18 (18.56)	9 (12.86)	16 (20.25)	0.465	74 (22.70)	25 (21.37)	28 (25.45)	21 (21.21)	0.699	87 ^22^ (27.36)	19 (31.67)	25 ^4^ (29.07)	43 (25.00)	0.559	0.013	0.161	0.039	0.561
Creatinine level [mg / dl]; (mean±SD)	1.38 ±1.09	1.24 ±0.86	1.26 ±0.75	1.33 ±1.29	1.13 ±0.35	0.371	1.29 ±1.00	1.28 ±0.76	1.34 ±1.29	1.25 ±0.89	0.166	1.59 ^222^ ±1.29	1.70 ^33 7^ ±1.37	1.51 ^8^ ±1.13	1.59 ^555 999^ ±1.34	0.416	<0.001	0.020	0.151	0.001
	System and history of pacing																			
HV lead presence before TLE n (%)	278 31.24)	74 (30.08)	32 (32.99)	28 (40.00)	14 ^EEE DDD^ (17.72)	0.009	98 (30.06)	38 (32.48)	34 (30.91)	26 (26.26)	0.595	106 (33.33)	25 (41.67)	35 ^E^ (40.70)	46 ^D E^ (26.74)	0.026	0.714	0.387	0.606	0.595
CS lead presence before TLE n (%)	176 (19.78)	36 (14.63)	17 (17.53)	10 (14.29)	9 (11.39)	0.518	58 (17.79)	31 (26.50)	15 ^E^ (13.64)	12 ^E^ (12.12)	0.009	82 ^22^ (25.79)	20 ^3^ (33.33)	23 (26.74)	39 (22.67)	0.261	0.002	0.047	0.046	0.019
Number of leads in the heart before TLE (mean ± SD)	2.08 ±0.80	1.97 ±0.73	2.12 ±0.74	1.87 ^E^ ±0.76	1.86 ^E^ ±0.66	0.026	2.16 ^2^ ±0.80	2.37 ±0.92	2.15 ^4^ ±0.75	1.94 ^EEE L^ ±0.62	<0.001	2.08 ±0.83	2.28 ±1.04	2.09 ±0.81	2.01 ±0.76	0.261	0.018	0.107	0.043	0.333
Presence of abandoned leads before TLE n (%)	123 (13.82)	26 (10.57)	15 (15.64)	5 (7.14)	6 (7.59)	0.132	56 (17.18)	32 (27.35)	16 ^E^ (14.55)	8 ^E^ (8.08)	<0.001	41 (12.98)	9 (15.00)	10 (11.63)	22 (12.79)	0.835	0.070	0.035	0.428	0.291
Number of previous CIED-related procedures (median, IQR)	2 [1–2]	2 [1–2]	2 [2–2]	2 ^EE^ [1–2]	2 ^EE^ [1–2]	0.015	2 [1–2]	2 [2–3]	2 [1–2]	1 ^EEE^ [1–2]	<0.001	1 ^2 66^ [1–2]	2 ^333^ [1–2]	2 [1–2]	1 ^E^ [1–2]	0.012	0.004	0.002	0.712	0.994
Dwell time of the oldest lead per patient during TLE [months] (mean ±SD)	91.64 ±69.65	92.39 ±68.86	89.59 ±58.71	72.23 ±70.32	113.7 ^DDD^ ±73.77	<0.001	90.45 ±69.93	97.69 ±74.91	81.52 ±75.53	91.82 ^D^ ±55.44	0.034	92.29 ±70.19	67.33 ^3 77^ ±64.63	85.62 ±84.79	104.3 ^EEE DDD^ ±61.07	<0.001	0.967	0.016	0.758	0.238
Dwell time of the oldest lead per patient during last procedure before TLE [months] (mean ±SD)	55.40 ±69.46	57.84 ±64.75	79.97 ±58.45	48.84 ^EEE^ ±70.28	38.39 ^EEE^ ±59.39	<0.001	62.28 ±73.46	91.47 ±76.66	57.83 ±75.34	33.00 ^EEE^ ±52.36	<0.001	46.35 ^22 66^ ±67.94	61.48 ^77^ ±65.15	61.19 ±84.70	33.52 ^EEE DDD^ ±6.24	<0.001	0.003	0.167	0.589	0.632
History of early CIED intervention n (%)	44 (4.94)	14 (5.69)	8 (8.25)	4 (5.71)	2 (2.53)	0.267	17 (5.21)	11 (9.40)	3 (2.73)	3 (3.09)	0.040	13 (4.09)	3 (5.00)	7 (8.14)	3 (1.74)	0.047	0.682	0.593	0.210	0.800
Large lead loop presence in X-Ray before TLE n (%)	46 (5.17)	8 (3.25)	3 (3.09)	1 (1.43)	4 (5.06)	0.457	14 (4.29)	5 (4.27)	6 (5.45)	3 (3.03)	0.690	24 ^2^ (7.55)	3 (5.00)	5 (5.81)	16 (9.30)	0.431	0.010	0.568	0.314	0.006
Leads on the both side of the chest n (%)	28 (3.15)	4 (1.63)	2 (2.06)	1 (1.43)	1 (1.27)	0.907	9 (2.76)	4 (3.42)	4 (3.64)	1 (1.01)	0.443	15 (4.72)	4 (6.67)	3 (3.49)	8 (4.65)	0.672	0.093	0.323	0.670	0.133
Lead abrasion; n (%)	225 (25.28)	56 (22.76)	22 (22.68)	19 (27.14)	15 (18.99)	0.614	66 (20.25)	31 (26.50)	14 ^4^ (12.73)	21 (21.21)	0.041	113 ^22 666^ (35.53)	21 (6.67)	29 ^888^ (33.72)	63 ^55 9^ (20.93)	0.840	<0.001	0.147	0.002	0.001
	The type of last procedure before TLE																			
Primary system implantation; n (%)	404 (45.39)	100 (40.65)	16 (16.49)	36 ^EEE^ (51.43)	48 ^EEE^ (60.76)	<0.001	133 (40.80)	17 (14.53)	55 ^EEE^ (50.00)	61 ^EEE^ (61.62)	<0.001	171 ^22 66^ (53.37)	21 ^7^ (35.00)	38 (44.19)	112 ^EEE DD^ (65.12)	<0.001	<0.001	0.003	0.712	0.893
Unit replacement; n (%)	307 (34.49)	102 (41.46)	58 (59.79)	21 ^EEE^ (30.00)	23 ^EEE^ (29.11)	<0.001	124 (38.04)	61 (52.14)	38 ^EE^ (34.55)	25 ^EE^ (25.25)	0.002	81 ^222 666^ (25.47)	23 ^3^ (38.33)	25 (29.07)	33 ^EE^ (19.19)	0.009	<0.001	0.030	0.645	0.381
Up-grading of the system; n (%)	57 (6.40)	16 (6.50)	7 (7.22)	7 (10.00)	2 (2.53)	0.172	20 (6.13)	8 (6.84)	7 (6.36)	5 (5.05)	0.856	21 (6.60)	4 (6.67)	11 (12.79)	6 ^D^ (3.49)	0.018	0.952	0.990	0.281	0.686
Any CIED-related procedure (excluding primary system implantation); n (%)	486 (54.61)	146 (59.34)	81 (83.51)	34 ^EEE^ (48.57)	31 ^EEE^ (39.24)	<0.001	193 (59.20)	100 (85.47)	55 ^EEE^ (50.00)	38 ^EEE^ (38.38)	<0.001	147 ^22 66^ (46.27	39 ^3 7^ (65.00)	48 (55.81)	60 ^EEE DD^ (34.88)	<0.001	<0.001	<0.001	0.809	0.893

Abbreviations: AF—atrial fibrillation, BMI—body mass index, CIED—cardiac implantable electronic device, CRT—cardiac resynchronization therapy, CS—coronary sinus, ICD—implantable cardioverter-defibrillator, HV—high voltage (lead), LVEF—left ventricular ejection fraction, NYHA—New York Heart Association, TLE—transvenous lead extraction. ^E^—*p* < 0.05, ^EE^—*p* < 0.01, ^EEE^—*p* < 0.001 (^E^—compared to early infection, ^D^—compared to delayed infection, ^L^—compared to late infection within the groups: PI, PI + LRIE, LRIE). ^N^—*p* < 0.05, ^NN^—*p* < 0.01, ^NNN^—*p* < 0.001 (compared to the data from column N). ^E^—early, ^D^—delayed, ^L^—late infectious complications.

Analysis of cultures from the pocket, leads or blood showed that coagulase-negative Staphylococci (CoNS) were more often found in early vs. delayed and late LRIE (6.67 vs. 5.81% vs. 1.16%; *p* = 0.05). Staphylococcus epidermidis was most common in patients with early PI + LRIE compared to early PI (28.21% vs. 12.37%; *p*= 0.018) and less common in delayed LRIE compared to delayed PI + LRIE (16.28% vs. 31.82%; *p*= 0.034). This pathogen was also less frequent in patients with late PI + LRIE compared to early and delayed infections of this type (14.14% vs. 28.21% vs. 31.82; *p* = 0.009). 

Staphylococcus aureus was most often found in patients with PI + LRIE, especially in the group of late PI + LRIE compared to late isolated LRIE (18.18% vs. 7.56%; *p* = 0.012). 

Negative cultures were observed more often in patients with early PI compared to LRIE + PI (35.77% vs. 20.51%; *p* = 0.014) and in the group of delayed LRIE compared to delayed LRIE + PI (32.56% vs. 16.36%; *p* = 0.01) (Table 3).

Multivariate Cox regression analysis showed that the main risk factor of isolated pocket infections in all analyzed time intervals was any CIED-related procedure before TLE, and the probability of PI occurrence was highest in the early period after the revision procedure (HR = 7.350; 5.507–10.865 95%CI; *p* < 0.001). The probability of PI in all analyzed time intervals also increased with the age of patients during the last procedure before TLE. The risk of late PI was lower in women (HR = 0.468; 0.284–0.769 95%CI; *p* = 0.003).

The probability of developing PI + LRIE increased with any CIED-related procedure, especially in the early period since the revision procedure (HR = 8.388; 6.070–11.591 95%CI; *p* < 0.001). The risk of PI + LRIE in all analyzed time intervals also increased with the patient’s age during the preceding procedure and was higher in patients with abandoned leads. The probability of early and delayed PI + LRIE increased in patients with a higher creatinine level. The risk of early, delayed and late PI+ LRIE was lower in patients with a shorter dwell time of the oldest lead. The probability of the delayed and late PI + LRIE was also lower in females and in patients that used long-term anticoagulation therapy.

The probability of occurrence of the isolated LRIE in all time intervals increased with any CIED-related procedure and was highest in the early period (HR-5.733. 95%CI: 3.662–8.975; *p* < 0.001). The risk of early, delayed and late LRIE was also higher in patients with abrasion of the leads. The probability of late LRIE increased in patients with a higher NYHA class and higher creatinine level (HR = 1.490. 95%CI: 1.123–1.978; *p* = 0.006 and HR = 1.270. 95%CI 1.137–1.418; *p* < 0.001). The risk of isolated LRIE in the late period after last procedure was also higher in patients with lead in the coronary sinus and with abandoned lead (HR = 2.322. 95%CI 1.471–3.664; *p* < 0.001 and HR = 2.210. 95%CI 1.213–4.029; *p* = 0.010). The probability of early LRIE was higher in patients with the leads on both sides of the chest (HR = 3.752; 95%CI 1.036–13.588; *p* = 0.044).

The probability of LRIE in all analyzed time intervals decreased in patients with a shorter dwell time of the oldest lead (Table 4).

The comparative analysis of the risk of early versus late infections showed that the probability of late infections increased in patients with a higher NYHA class (OR = 1.024. 95% CI 1.008–1.038; *p* = 0.002). Risk of early infectious complications increased with the age of patients and with the body mass index (BMI) (OR = 1.654. 95% CI 1.213–1.256, *p* < 0.001, and OR = 1.053. 95% CI 1.004–1.103, *p* = 0.032, respectively). 

With regard to device-related factors, patients with more leads (OR = 2.387. 95% CI 1.188- 4.785; *p* = 0.014) presented a higher probability of late infections. Patients with high voltage (HV) leads (OR = 3.040. 95% CI 1.761–5.236; *p* < 0.01), longer lead dwell time during last revision procedure (OR = 1.091. 95% CI 1.031–1.153; *p* = 0. 03), any previous CIED-related procedure (OR = 4.016. 95% CI 2.304–6.993; *p* < 0.01) and the presence of CoNS in cultures (OR = 3.994. 95% CI 1.344–11.776; *p* = 0.012) were more likely to have early rather than late infection. The probability of early compared to late PI was also higher in patients with CRT-D (OR = 7.299. 95% CI 1.078–50.00; *p* = 0.040).

Patients with more leads were more likely to develop late PI + LRIE, but the tendency was only seen in the univariate analysis, whereas an increased risk of early versus late PI + LRIE was observed after any CIED-related procedure and in patients with the presence of Staphylococcus epidermidis in cultures (OR = 2.326. 95% CI 0.993–5.435; *p* = 0.051).

The probability of early LRIE was higher in patients with HV leads (OR = 3.367. 95% CI 1.401–8.130; *p* = 0.006), with older leads during last CIED-related procedure (OR = 2.326 95% CI 0.993–5.435; *p* = 0.051) and with the presence of CoNS in cultures (OR = 10.00 95% CI 1.429–71.43; *p* = 0.020) (Table 5).

**Figure 2:**
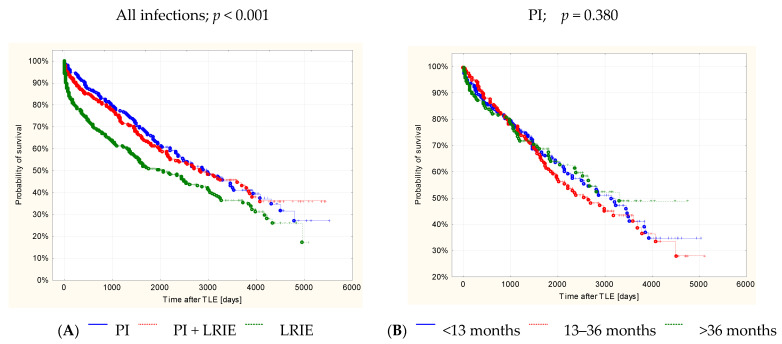

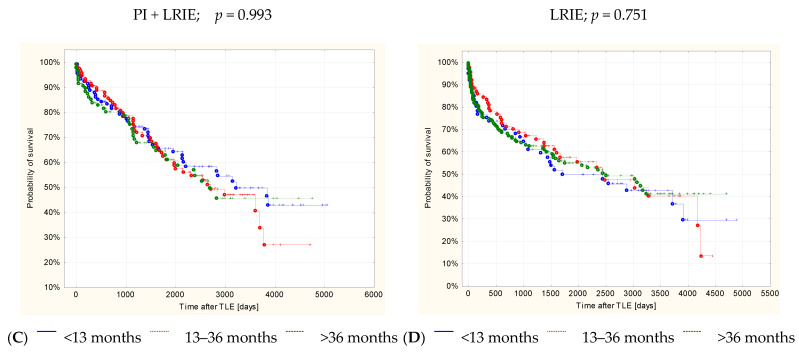
Probability of survival depending on onset of infection: (**A**) All cardiac implantable device infections. (**B**) Isolated pocket infections. (**C**) Pocket infection coexisting with systemic infections. (**D**) Lead-related infective endocarditis. (**A**): Probability of survival after TLE depending on infection type *p* < 0.001. (**B**): Probability of survival after TLE due to pocket infection depending on temporal onset related to last CIED procedure *p* = 0.680. (**C**): Probability of survival after TLE due to pocket and systemic infection depending on temporal onset related to last CIED procedure *p* = 0.993. (**D**): Probability of survival after TLE due to systemic infection depending on temporal onset related to last CIED procedure *p* = 0.751.

At long-term follow-up after TLE (1826± 1381 days), the probability of event-free survival at all time intervals was highest in patients with PI (about 45% after 10 years) and lowest in those with LRIE (about 35% after 10 years) (Figure 2A). Detailed analysis of each group: isolated PI (Figure 2B), PI + LRIE (Figure 2C) and isolated LRIE (Figure 2D) showed no significant differences in survival rates between successive time intervals. 

## 4. Discussion

Infectious complications in patients with cardiac implantable electronic devices occur at various times after initial placement and appear to be frequently associated with previous device procedures. Prior studies have shown that rates of infection were two to five times higher after revision procedures than after initial implantation (initial 0.5–0.8%, revision 1–4% at 1 to 3 years of follow-up) [15,16,17]. In the present study, the rate of cardiac device infection after a CIED-related procedure was only slightly higher than that observed after initial implantation (54.61% vs. 45.39%), and the most common infectious complications were found up to 12 months after generator replacement procedures. Multi-year analysis of 62 infection cases documented similar results: 33 (53.2%) infections occurred after initial implantation, 18 (29.0%) after the second procedure and 11 (17.7%) after the third and more procedures [18]. Previous reports comparing early and late CIED infections showed that the symptoms of pocket infection (erythema, pain, swelling, warmth and pus or drainage from the pocket) were likely to occur 6 to 12 months since last device procedure, whereas the signs of systemic symptoms predominated in late infections [7,19,20,21]. The current study confirmed a relatively high incidence of PI in the early period after the preceding procedure, while LRIE was more frequent in more than 36 months after the last CIED-related procedure. However, the present study demonstrated that a high rate of PI also occurred at >12 months after any revision procedure (over 50% of infectious complications, including 23.26% of PI occurring at >60 months after last CIED procedure). The same tendency was already observed in one previous report [7]; however, the factors that may favor the development of late pocket infections without a direct relationship with previous CIED procedure have not been identified so far. It was postulated that the risk of late PI was higher in patients with a too-shallow generator location and, consequently, progressive erosion of the pocket, as well as with the identification of less virulent pathogens, mainly coagulase-negative Staphylococci [7]. 

The spread of an infection from the device pocket along the leads to the endocardium is, according to most investigators, the most likely cause of LRIE (systemic infection) [7,19,20,21,22,23,24,25,26,27]. In the present study, PI + LRIE occurred at a similar rate at all time intervals, whereas isolated LRIE was more evident over an extended period of time (>50% of LRIE cases occurred at >5 years after last procedure). Moreover, the present study revealed that the probability of early versus late LRIE increased with the presence of CoNS, which supports the concept of bacteria spreading from the infected pocket along the leads to the endocardium. However, such a coincidence was confirmed only for early LRIE, especially coexisting with PI, whereas late systemic infections, most common, were associated with the process of intracardiac lead abrasion. The influence of lead abrasion on the development of lead-related infective endocarditis was described in our previous reports [14,28,29,30], whereas the current study confirmed that the pathogenesis of late isolated LRIE was different and probably not directly related to bacterial contamination of the pocket. This explanation may be supported by the higher incidence of Staphylococcus aureus in patients with delayed systemic infections in this study. Moreover, we demonstrated that the development of late LRIE was influenced by clinical factors such as heart failure and renal failure. Probably, reduced immunity increases the risk of infection, and externalized conductors due to abrasion-related breach of the insulation facilitate the migration of pathogens along the lead and formation of biofilm.

Long-term follow-up of patients undergoing TLE for infectious indications showed high mortality in patients with systemic infections. The relationship between time to infection onset and long-term survival has not been established. Due to the high mortality rate of patients with CIED-related infections, it is important to minimize infectious complications. In order to reduce the risk of infection, antibacterial envelopes, leadless pacemakers and subcutaneous ICD systems are increasingly used. Current research confirms the good effects of new technologies. The use of leadless pacemakers allows for the complete elimination of the risk of pocket infection. Recent studies confirm that by improving the leadless pacemaker implantation technique, electrical stability is ensured, which reduces the need for reintervention [31]. In patients requiring ICD implantation, the use of subcutaneous s-ICD eliminates the occurrence of LRIE, and according to recent studies, the risk of device-related complications related to s-ICD during the 2-year follow-up period is relatively rare [32].

## 5. Limitations

This is a post hoc analysis, and therefore no randomization was performed. Our findings are limited by the ability to analyze only the patient population undergoing TLE.

## 6. Conclusions

Most cardiac device infections occur more than 12 months after the preceding CIED-related procedure. The occurrence of various types of infections at different time intervals after last revision procedure is associated with specific risk factors. Early PI is more often associated with last CIED procedure, whereas the risk of LRIE, especially late after last revision procedure, is higher in patients with intracardiac lead abrasion. The pathogenesis of PI + LRIE is probably related to the contamination of device pocket with low-virulent pathogens (most often CoNS) during initial implantation or during previous CIED procedure. The timing of infection onset irrespective of its type does not affect long-term survival after transvenous lead extraction. The persistently high mortality of patients with CIED-related infections requires the search for methods to minimize the risk of infectious complications.

## Figures and Tables

**Table 3:** Distribution of culture pathogens depending on type of infection and temporal onset related to last CIED procedure for noninfectious indications.

			Early (E)	Delayed (D)	Late (L)	ANOVA 3–5		Early (E)	Delayed (D)	Late (L)	ANOVA 7–9		Early (E)	Delayed (D)	Late (L)	ANOVA				
	1	2	3	4	5		6	7	8	9		10	11	12	13	(11 12 13)	(2 6 10)	(3 7 11)	(4 8 12)	(5 9 13)
	All pts	PI (All)	0–12 month.	13–36 month	>36 month	*p*	PI + LRIE (All)	0–12 month	13–36 month	>36 month	*p*	LRIE (All)	0–12 month	13–36 month	>36 month	*p*	*p*	*p*	*p*	*p*
Number of patients (n, %)	890	246 (100.0)	97 (39.43)	70 (28.46)	79 (32.1)		326 (100.0)	117 (35.89)	110 (33.74)	99 (30.37)		318 (100.0)	60 (18.87)	86 (27.04)	172 (54.09)					
CoNS; n (%)	40 (4.49)	16 (6.50)	6 (6.91)	8 (11.43)	2 (2.53)	0.890	13 (3.99)	5 (4.27)	5 (4.55)	3 (3.03)	0.839	11 (3.46)	4 (6.67)	5 (5.81)	2 (1.16)	0.050	0.669	0.747	0.190	0.669
Staph epidermidis; n (%)	183 (20.56)	39 (15.85)	12 (12.37)	14 (20.00)	13 (16.46)	0.407	82 (25.15)	33 ^E 33^ (28.21)	35 (31.82)	14 ^E DD^ (14.14)	0.009	62 (19.50)	12 (20.00)	14 ^D 8^ (16.28)	36 (20.93)	0.670	0.450	0.018	0.034	0.449
Staph aureus; n (%)	102 (11.46)	20 (8.13)	11 11.44)	4 (5.71)	5 (6.33)	0.330	54 ^22^ (16.56)	20 (17.09)	16 (14.55)	18 ^E 5^ (18.18)	0.766	28 ^66^ (8.81)	8 (13.33)	7 (8.14)	13 ^L 99^ (7.56)	0.386	0.012	0.476	0.928	0.012
Staph—other; n (%)	11 (1.24)	6 (2.44)	4 (4.12)	2 (2.86)	0 (0.00)	0.205	5 (1.53)	2 (1.71)	1 (0.91)	2 (2.02)	0.794	0 (0.00)	0 (0.00)	0 (0.00)	0 (0.00)	1.000	0.154	0.207	0.241	0.154
STI n (%)	60 (6.74)	10 (4.07)	2 (2.06)	4 (5.71)	4 (5.06)	0.431	22 (6.75)	4 (3.42)	11 (10.00)	7 (7.07)	0.141	28 8.81)	5 (8.33)	9 (10.47)	14 (8.14)	0.817	0.669	0.138	0.512	0.669
Streptococcus; n (%)	3 (0.34)	0 (0.00)	0 (0.00)	0 (0.00)	0 (0.00)	1.000	1 (0.31)	0 (0.00)	1 (0.91)	0 (0.00)	0.375	2 (0.63)	0 (0.00)	0 (0.00)	2 (1.16)	0.427	0.514	1.00	0.497	0.514
Other bacteria; n (%)	64 (7.19)	10 (4.07)	6 (6.91)	3 (4.29)	1 (1.27)	0.259	23 (7.06)	10 (8.55)	5 (4.55)	8 (8.08)	0.448	31 (9.75)	6 (10.00)	9 (10.47)	16 (9.30)	0.955	0.077	0.670	0.155	0.077
Culture negative; n (%)	243 (27.30)	88 (35.77)	37 (38.14)	23 (32.86)	28 (35.44)	0.780	70 (21.47)	24 ^EE 33^ (20.51	18 ^E 4^ (16.36)	28 (28.28)	0.108	85 (26.73)	15 (25.00)	28 ^D 88^ (32.56)	42 (24.42)	0.358	0.137	0.014	0.010	0.137
Lack of culture result; n (%)	184 (20.67)	57 (23.17)	19 (19.59)	12 (17.14)	26 (32.91	0.043	56 (17.18)	19 (16.24)	18 (16.36	19 (19.19)	0.817	71 (22.33)	10 (16.67)	14 (16.28)	47 (27.33)	0.068	0.340	0.799	0.962	0.340

Abbreviations: CoNS—coagulase-negative Staphylococci, LRIE—lead-related infective endocarditis, PI—pocket infection, STI—Staphylococcus auricularis. ^E^—*p* < 0.05, ^EE^—*p* < 0.01 (^E^—compared to early infection, ^D^—compared to delayed infection, ^L^—compared to late infection within the groups: PI, PI + LRIE, LRIE). ^N^—*p* < 0.05, ^N^^N^—*p* < 0.01 (compared to the data from column N). ^E^—early, ^D^—delayed, ^L^—late infectious complications.

**Table 4:** Multivariate Cox regression analysis of risk factors of occurrence of different types of CIED-related infections in particular time intervals.

Multivariate Cox Regression Analysis									
Parameters	12 Months Follow-Up			36 Months Follow-Up			Over 36 Months Follow-Up		
	HR	95% CI	*p*	HR	95% CI	*p*	HR	95% CI	*p*
**Isolated PI**									
	**n = 2929**			**n = 2642**			**n = 2307**		
Patient’s age during last procedure before TLE (years)	1.035	1.015–1.055	0.001	1.028	1.006–1.051	0.013	1.042	1.020–1.066	<0.001
Sex (% of female patients) (%)	1.008	0.647–1.571	0.972	0.295	0.151–0.574	0.000	0.468	0.284–0.769	0.003
AF permanent	1.287	0.806–2.054	0.291	1.529	0.914–2.557	0.106	1.016	0.625–1.649	0.950
Device type—CRT-D	1.130	0.467–2.734	0.787	1.174	0.431–3.193	0.754	0.631	0.135–2.954	0.559
Number of leads in the system before TLE	1.054	0.716–1.552	0.788	0.922	0.599–1.417	0.710	0.948	0.618–1.453	0.806
HV lead presence before TLE	0.898	0.505–1.595	0.713	1.076	0.589–1.967	0.811	1.162	0.593–2.276	0.663
Early CIED intervention in history	1.210	0.570–2.567	0.619	1.129	0.343–3.721	0.841	1.069	0.260–4.396	0.926
Dwell time of the oldest lead in the patient before TLE	0.993	0.989–0.996	0.000	0.986	0.980–0.991	0.000	0.994	0.990–0.998	0.002
Any CIED-related procedure	7.735	5.507–10.865	0.000	4.294	2.837–6.501	0.000	3.863	2.513–5.936	<0.001
**PI + LRIE**									
	**n = 2503**			**n = 2642**			**n = 2307**		
Patient’s age during last procedure before TLE (years)	1.018	1.001–1.036	0.043	1.028	1.010–1.047	0.002	1.048	1.027–1.071	<0.001
Sex (% of female patients) (%)	0.862	0.569–1.304	0.481	0.593	0.383–0.919	0.019	0.452	0.280–0.728	0.001
LVEF (%)	1.410	0.806–2.469	0.229	1.304	0.699–2.433	0.403	1.325	0.688–2.554	0.400
Diabetes (any)	1.007	0.632–1.605	0.977	1.369	0.874–2.144	0.170	1.024	0.622–1.686	0.926
Creatinine level (mg%)	1.262	1.026–1.552	0.028	1.233	1.035–1.468	0.019	1.124	0.881–1.434	0.348
Long-term anticoagulation therapy	0.696	0.421–1.152	0.159	0.484	0.280–0.835	0.009	0.514	0.285–0.929	0.027
Number of leads in the system before TLE	1.414	0.946–2.113	0.091	1.655	1.083–2.532	0.020	1.109	0.701–1.756	0.658
Presence of abandoned lead before TLE	3.216	1.886–5.486	0.000	2.294	1.164–4.519	0.016	3.183	1.342- 7.548	0.009
HV lead presence before TLE	0.812	0.509–1.295	0.381	0.916	0.577–1.453	0.709	1.099	0.644–1.876	0.729
CS lead presence before TLE	0.951	0.512–1.769	0.875	0.433	0.207–0.907	0.026	0.819	0.375–1.787	0.616
Early CIED intervention in history	1.447	0.732–2.859	0.288	0.653	0.202–2.107	0.476	0.936	0.281–3.123	0.915
Upgrading or additional lead implantation	1.346	0.806–2.245	0.256	1.701	0.930–3.110	0.084	0.265	0.091–0.765	0.014
Dwell time of the oldest lead before TLE	0.994	0.991–0.998	0.001	0.989	0.985–0.993	0.000	0.985	0.980–0.991	<0.001
Any CIED-related procedure	8.388	6.070–11.591	0.000	4.619	3.271–6.523	0.000	4.276	2.851–6.414	<0.001
**Isolated LRIE**									
	**n = 2693**			**n = 2462**			**n = 2216**		
Patient’s age during last procedure before TLE (years)	1.014	0.988–1.041	0.289	1.029	1.008–1.051	0.007	1.004	0.989–1.018	0.622
Female gender	0.896	0.481–1.669	0.730	0.901	0.542–1.497	0.687	0.666	0.466–0.954	0.027
NYHA I-IV	1.050	0.652–1.693	0.840	0.857	0.565–1.300	0.467	1.490	1.123–1.978	0.006
LVEF	1.015	0.990–1.040	0.241	0.988	0.970–1.007	0.228	1.008	0.995–1.021	0.230
Diabetes (any)	1.806	0.986–3.307	0.056	1.675	1.016–2.761	0.043	1.296	0.886–1.896	0.181
Creatinine level (mg%)	1.244	1.065–1.452	0.006	1.164	0.986–1.374	0.073	1.270	1.137–1.418	0.000
Long-term antiplatelet therapy	0.746	0.412–1.351	0.334	0.856	0.528–1.387	0.527	0.966	0.680–1.370	0.844
Number of leads in the system	1.379	0.823–2.312	0.222	1.061	0.697–1.613	0.783	0.741	0.538–1.019	0.065
HV lead presence before TLE	1.187	0.588–2.397	0.633	1.474	0.839–2.589	0.177	1.405	0.911–2.167	0.124
CS lead presence before TLE	1.505	0.691–3.281	0.304	1.219	0.641–2.321	0.546	2.322	1.471–3.664	<0.001
Lead implanted before age of 20	3.466	0.635–18.923	0.151	6.105	1.563–23.837	0.009	0.658	0.199–2.170	0.491
Presence of abandoned lead before TLE	1.458	0.536–3.970	0.460	1.360	0.610–3.030	0.452	2.210	1.213–4.029	0.010
Abrasion of the lead	2.603	1.371–4.943	0.003	2.402	1.428–4.041	0.001	2.610	1.792–3.801	<0.001
Leads on both sides of the chest before TLE	3.752	1.036–13.588	0.044	1.923	0.533–6.945	0.318	1.387	0.538–3.578	0.499
Dwell time of the oldest lead before TLE	0.982	0.976–0.988	0.000	0.987	0.982–0.992	0.000	0.978	0.973–0.983	<0.001
Any CIED-related procedure	5.733	3.662–8.975	0.000	5.497	3.768–8.019	0.000	5.230	3.890–7.030	<0.001

Abbreviations: AF—atrial fibrillation, CIED—cardiac implantable electronic device, CRT—cardiac resynchronization therapy, CS—coronary sinus, HV—high voltage, LRIE—lead-related infective endocarditis, PI—pocket infection, LVEF—left ventricular ejection fraction, NYHA—New York Heart Association, TLE—transvenous lead extraction.

**Table 5:** Clinical, procedural, bacteriological and CIED-related factors affecting onset (<12 months or >36 months) of infections after revision procedure. The results of univariable and multivariable linear regression analysis.

Infectious Complications (All)							
			Univariable			Multivariable	
		OR	95% CI	*p*	OR	95% CI	*p*
E	Patient age during the last procedure before TLE (one year)	1.018	1.007–1.031	0.002	1.024	1.008–1.038	0.002
L	NYHA (one class)	1.269	1.012–1.591	0.039	1.654	1.213–2.256	<0.001
E	BMI (one unit)	1.043	1.003–1.082	0.033	1.053	1.004–1.103	0.032
E	CRT-D (yes/no)	3.040	1.718–5.376	<0.001	1.451	0.545–3.861	0.456
E	Number of leads in the system before TLE (by one)	1.650	1.282–2.123	<0.001	2.387	1.188–4.785	0.014
E	Number of leads in the heart before TLE (by one)	1.618	1.312–1.992	<0.001	0.741	0.398–1.377	0.343
E	Abandoned leads (yes/no)	2.169	1.376–3.425	<0.001	1.524	0.596–3.891	0.378
E	HV lead before TLE (yes/no)	1.493	1.057–2.110	0.023	3.040	1.761–5.236	<0.001
E	CS lead before TLE (yes/no)	1.613	1.087–2.392	0.017	0.535	0.270–1.059	0.072
E	Number of previous procedures (by one)	1.946	1.608–2.347	<0.001	0.896	0.682–1.178	0.430
E	History of early CIED intervention (yes/no)	3.704	1.618–8.475	0.002	2.137	0.852–5.348	0.105
E	Upgrading or additional lead implantation (yes/no)	2.500	1.572–3.968	<0.001	1.147	0.639–2.058	0.646
E	Dwell time of oldest lead per patient during the last procedure before TLE (by one year)	1.157	1.117–1.200	<0.001	1.091	1.031–1.153	0.003
E	Any procedure other than first system implantation (yes/no)	6.711	4.651–9.709	<0.001	4.016	2.304–6.993	<0.001
E	Presence of CoNS (yes/no)	2.817	1.129–6.993	0.026	3.984	1.344–11.776	0.012
Isolated pocket infection							
E	CRT-D (yes/no)	5.376	1.151–25.00	0.031	7.299	1.078–50.00	0.040
E	Number of leads in the system before TLE (by one)	1.695	1.012–2.833	0.043	1.271	0.455–3.546	0.645
E	Number of leads in the heart before TLE (by one)	1.650	1.043–2.611	0.031	0.765	0.316–1.848	0.549
E	Number of previous procedures (by one)	2.141	1.397–3.279	<0.001	0.768	0.440–1.340	0.350
E	Dwell time of oldest lead per patient during the last procedure before TLE (by one year)	1.166	1.085–1.253	<0.001	1.003	0.995–1.011	0.477
E	Any procedure other than first system implantation (by one)	8.547	4.202–17.54	<0.001	10.00	3.378–29.41	<0.001
Pocket infection with concomitant infective endocarditis							
E	CRT-D (yes/no)	2.513	1.056–5.988	0.036	1.427	0.233–8.772	0.699
E	Number of leads in the system before TLE (by one)	1.812	1.160–2.833	0.009	2.538	0.608–10.64	0.199
L	Number of leads in the heart before TLE (by one)	2.092	1.425–3.077	<0.001	0.583	0.162–2.101	0.406
E	Abandoned leads (yes/no)	4.329	1.880–10.00	<0.001	1.815	0.244–13.51	0.558
E	CS leads before TLE (yes/no)	2.646	1.269–5.495	0.009	0.839	0.165–4.274	0.832
E	Number of previous procedures (by one)	3.279	2.174–4.926	<0.001	1.506	0.858–2.646	0.152
E	Upgrading or additional lead implantation (yes/no)	6.849	2.532–18.52	<0.001	3.745	0.653–21.28	0.136
E	Upgrading or downgrading with lead abandonment (yes/no)	5.495	1.550–19.61	0.008	0.479	0.044–5.208	0.543
E	Dwell time of oldest lead per patient during the last procedure before TLE (by one year)	1.203	1.126–1.287	<0.001	1.053	0.955–1.160	0.301
E	Any procedure other than first system implantation (yes/no)	9.091	4.762–17.54	0.000	3.155	1.285–7.752	0.012
E	Staph epidermidis (yes/no)	2.415	1.200–4.854	0.013	2.326	0.993–5.435	0.051
Isolated lead-related infective endocarditis							
L	CRT-D (yes/no)	0.366	0.133–1.002	0.049	1.366	0.305–6.117	0.682
E	Number of leads in the system before TLE (by one)	1.661	1.086–2.545	0.019	2.037	0.929–4.464	0.074
L	Number of leads in the heart before TLE (by one)	0.692	0.491–0.974	0.034	1.128	0.573–2.218	0.726
E	HV leads before TLE (by one)	1.908	1.029–3.546	0.039	3.367	1.401–8.130	0.006
L	Number of previous procedures (by one)	0.766	0.593–0.989	0.040	1.357	0.808–2.280	0.246
E	Dwell time of oldest lead per patient during the last procedure before TLE (by one year)	1.091	1.030–1.153	0.003	1.110	1.000–1.232	0.049
E	Any procedure other than first system implantation (yes/no)	3.058	1.653–5.650	<0.001	2.475	0.895–6.849	0.079
E	Presence of CoNS (yes/no)	5.952	1.054–33.33	0.042	10.00	1.429–71.43	0.020

Abbreviations: BMI—body mass index, CIED—cardiac implantable electronic device, CoNS—coagulase-negative Staphylococci, CRT—cardiac resynchronization therapy, CS—coronary sinus, HV—high voltage, NYHA—New York Heart Association, TLE—transvenous lead extraction.

## Data Availability

Data are contained within the article.

## References

[1] Clementy N., Carion P.L., de Leotoing L., Lamarsalle Wilquin-Bequet F., Brown B., Verhees K.J.P., Fernandes J., Deharo J.C. (2018). Infections and associated costs following cardiovascular implantable electronic device implantations: A nationwide cohort study. Europace.

[2] Ludwig S., Theis C., Brown B., Witthohn A., Lux W., Goette A. (2018). Incidence and costs of cardiac device infections: Retrospective analysis using German health claims data. J. Comp. Eff. Res..

[3] Sandoe J.A., Barlow G., Chambers J.B., Gammage M., Guleri A., Howard P., Olson E., Perry J.D., Prendergast B.D., Spry M.J. (2015). Guidelines for the diagnosis, prevention and management of implantable cardiac electronic device infection. Report of a joint Working Party project on behalf of the British Society for Antimicrobial Chemotherapy (BSAC, host organization), British Heart Rhythm Society (BHRS), British Cardiovascular Society (BCS), British Heart Valve Society (BHVS) and British Society for Echocardiography (BSE). J. Antimicrob. Chemother..

[4] Uslan D.Z., Sohail M.R., St Sauver J.L., Friedman P.A., Hayes D.L., Stoner S.M., Wilson W.R., Steckelberg J.M., Baddour L.M. (2007). Permanent pacemaker and implantable cardioverter defibrillator infection: A population-based study. Arch. Intern. Med..

[5] Baddour L.M., Epstein A.E., Erickson C.C., Knight B.P., Levison M.E., Lockhart P.B., Masoudi F.A., Okum E.J., Wilson W.R., Beerman L.B. (2010). Update on cardiovascular implantable electronic device infections and their management: A scientific statement from the American Heart Association. Circulation.

[6] Korkerdsup T., Ngarmukos T., Sungkanuparph S., Phuphuakrat A. (2018). Cardiac implantable electronic device infection in the cardiac referral center in Thailand: Incidence, microbiology, risk factors, and outcomes. J. Arrhythm..

[7] Welch M., Uslan D.Z., Greenspon A.J., Sohail M.R., Baddour L.M., Blank E., Carrillo R.G., Danik S.B., Del Rio A., Hellinger W. (2014). Variability in Clinical Features of Early Versus Late Cardiovascular Implantable Electronic Device Pocket Infections. Pacing Clin. Electrophysiol..

[8] Wang R., Li X., Wang Q., Zhang Y., Wang H. (2017). Microbiological Characteristics and Clinical Features of Cardiac Implantable Electronic Device Infections at a Tertiary Hospital in China. Front. Microbiol..

[9] Habib G., Lancellotti P., Antunes M.J., Bongiorni M.G., Casalta J.P., Del Zotti F., Dulgheru R., El Khoury G., Erba P.A., Iung B. (2015). ESC guidelines for the management of infective endocarditis: The Task Force for the Management of Infective Endocarditis of the European Society of Cardiology (ESC). Endorsed by: European Association for Cardio-Thoracic Surgery (EACTS), the European Association of Nuclear Medicine (EANM). Eur. Heart J..

[10] Blomström-Lundqvist C., Traykov V., Erba P.A., Burri H., Nielsen J.C., Bongiorni M.G., Poole J., Boriani G., Costa R., Deharo J.C. (2020). European Heart Rhythm Association (EHRA) international consensus document on how to prevent, diagnose, and treat cardiac implantable electronic device infec-tions-endorsed by the Heart Rhythm Society (HRS), the Asia Pacific Heart Rhythm Society (APHRS), the Latin American Heart Rhythm Society (LAHRS), International Society for Cardiovascular Infectious Diseases (ISCVID), and the European Society of Clinical Microbiology and Infectious Diseases (ESCMID) in collaboration with the European Association for Cardio-Thoracic Surgery (EACTS). Eur. Heart J..

[11] Wilkoff B.L., Love C.J., Byrd C.L., Bongiorni M.G., Carrillo R.G., Crossley GH 3rd Epstein L.M., Friedman R.A., Kennergren C.E., Mitkowski P., Schaerf R.H. (2009). Transvenous Lead Extraction: Heart Rhythm Society Expert Consensus on Facilities, Training, Indications, and Patient Management. Heart Rhythm.

[12] Kusumoto F.M., Schoenfeld M.H., Wilkoff B.L., Berul C.I., Birgersdotter-Green U.M., Carrillo R., Cha Y.-M., Clancy J., Deharo J.-C., Ellenbogen K.A. (2017). 2017 HRS expert consensus statement on cardiovascular implantable electronic device lead management and extraction. Heart Rhythm.

[13] Bongiorni M.G., Burri H., Deharo J.C., Starck C., Kennergren C., Saghy L., Rao A., Tascini C., Lever N., Kutarski A. (2018). 2018 EHRA expert consensus statement on lead extraction: Recommendations on definitions, endpoints, research trial design, and data collection requirements for clinical scientific studies and registries: Endorsed by APHRS/HRS/LAHRS. Europace.

[14] Kutarski A., Małecka B., Kołodzinska A., Grabowski M. (2013). Mutual Abrasion of Endocardial Leads: Analysis of Explanted Leads. Pacing Clin. Electrophysiol..

[15] Catanchin A., Murdock C.J., Athan E. (2007). Pacemaker Infections: A 10-Year Experience. Heart Lung Circ..

[16] Nery P.B., Fernandes R., Nair G.M., Sumner G.L., Ribas C.S., Menon S.M.D., Wang X., Krahn A.D., Morillo C.A., Connolly S.J. (2010). Device-Related Infection Among Patients with Pacemakers and Implantable Defibrillators: Incidence, Risk Factors, and Consequences. J. Cardiovasc. Electrophysiol..

[17] Sohail M.R., Uslan D.Z., Khan A.H., Friedman P.A., Hayes D.L., Wilson W.R., Steckelberg J.M., Jenkins S.M., Baddour L.M. (2008). Infective Endocarditis Complicating Permanent Pacemaker and Implantable Cardioverter-Defibrillator Infection. Mayo Clin. Proc..

[18] Dai M., Cai C., Vaibhav V., Sohail M.R., Hayes D.L., Hodge D.O., Tian Y., Asirvatham R., Cochuyt J.J., Huang C. (2019). Trends of Cardiovascular Implantable Electronic Device Infection in 3 Decades: A Population-Based Study. JACC Clin. Electrophysiol..

[19] Greenspon A.J., Prutkin J.M., Sohail M.R., Vikram H.R., Baddour L.M., Danik S.B., Peacock J., Falces C., Miro J.M., Blank E. (2012). Timing of the Most Recent Device Procedure Influences the Clinical Outcome of Lead-Associated Endocarditis: Results of the MEDIC (Multicenter Electrophysiologic Device Infection Cohort). J. Am. Coll. Cardiol..

[20] Klug D., Lacroix D., Savoye C., Goullard L., Grandmougin D., Hennequin J.L., Kacet S., Lekieffre J. (1997). Systemic Infection Related to Endocarditis on Pacemaker Leads. Circulation.

[21] Ursaru A.M., Haba C.M., Popescu E., Crișu D., Petriș A.O., Tesloianu N.D. (2021). A Rare Entity–Percutaneous Lead Extraction in a Very Late Onset Pacemaker Endocarditis: Case Report and Review of Literature. Diagnostics.

[22] Machino T., Sekiguchi Y. (2015). Positive Pocket Cultures From Cardiac Implantable Electrophysiological Devices Without Infection—Contamination or Colonization?. Circ. J..

[23] Pichlmaier M., Marwitz V., Kühn C., Niehaus M., Klein G., Bara C., Haverich A., Abraham W.R. (2008). High prevalence of asymptomatic bacterial colonization of rhythm management devices. Europace.

[24] Kleemann T., Becker T., Strauss M., Dyck N., Weisse U., Saggau W., Burkhardt U., Seidl K. (2010). Prevalence of bacterial colonization of generator pockets in implantable cardioverter defibrillator patients without signs of infection undergoing generator replacement or lead revision. Europace.

[25] Rohacek M., Weisser M., Kobza R., Schoenenberger A.W., Pfyffer G.E., Frei R., Erne P., Trampuz A. (2010). Bacterial Colonization and Infection of Electrophysiological Cardiac Devices Detected with Sonication and Swab Culture. Circulation.

[26] Mason P.K., Dimarco J.P., Ferguson J.D., Mahapatra S., Mangrum J.M., Bilchick K.C., Wiggins D., Mounsey J.P., Moorman J.R. (2011). Sonication of explanted cardiac rhythm management devices for the diagnosis of pocket infections and asymptomatic bacterial colonization. Pacing Clin. Electrophysiol..

[27] Okada M., Kashiwase K., Hirata A., Nemoto T., Matsuo K., Murakami A., Ueda Y. (2015). Bacterial Contamination During Pacemaker Implantation Is Common and Does Not Always Result in Infection. Circ. J..

[28] Kołodzińska A., Kutarski A., Koperski Ł., Grabowski M., Małecka B., Opolski G. (2012). Differences in encapsulating lead tissue in patients who underwent transvenous lead removal. Europace.

[29] Kołodzińska A., Kutarski A., Kozłowska M., Grabowski M., Marchel H., Drela N., Opolski G. (2013). Biodegradation of the outer silicone insulation of endocardial leads. Circ. Arrhythm. Electrophysiol..

[30] Polewczyk A., Jacheć W., Janion M., Podlaski R., Kutarski A. (2015). Lead-Dependent Infective Endocarditis: The Role of Factors Predisposing to Its Development in an Analysis of 414 Clinical Cases. Pacing Clin. Electrophysiol..

[31] Mitacchione G., Arabia G., Schiavone M., Cerini M., Gasperetti A., Salghetti F., Bontempi L., Viecca M., Curnis A., Forleo G.B. (2022). Intraoperative sensing increase predicts long-term pacing threshold in leadless pacemakers. J. Interv. Card. Electrophysiol..

[32] Gasperetti A., Schiavone M., Ziacchi M., Vogler J., Breitenstein A., Laredo M., Palmisano P., Ricciardi D., Mitacchione G., Compagnucci P. (2021). Long-term complications in patients implanted with subcutaneous implantable cardioverter-defibrillators: Real-world data from the extended ELISIR experience. Heart Rhythm.

